# Therapeutic Efficacy of Dual-Targeting Nanoparticles with Low Immunogenicity in the Treatment of Rheumatoid Arthritis

**DOI:** 10.3390/jfb17050228

**Published:** 2026-05-06

**Authors:** Renjie Miao, Haoyu Wang, Yitian Jin, Changsheng Liu, Hongyan He

**Affiliations:** 1 Frontiers Science Center for Materiobiology and Dynamic Chemistry, East China University of Science and Technology, Shanghai 200237, China; miaorj0611@163.com (R.M.); liucs@ecust.edu.cn (C.L.); 2 School of Materials Science and Engineering, East China University of Science and Technology, Shanghai 200237, China; wanghaoyunh@163.com (H.W.); y30230900@mail.ecust.edu.cn (Y.J.); 3 Engineering Research Center for Biomedical Materials of Ministry of Education, East China University of Science and Technology, Shanghai 200237, China

**Keywords:** rheumatoid arthritis, dual-targeted nanoparticles, methotrexate, red blood cell membrane

## Abstract

Rheumatoid arthritis (RA) treatment is severely hindered by the systemic toxicity and limited joint accumulation of conventional therapeutics. To overcome these critical clinical challenges, we engineered a biomimetic dual-targeted nanoplatform (MTX@HSA@M@HA NPs) to precisely deliver methotrexate (MTX) to inflamed synovia. The rationally designed system encapsulates MTX within human serum albumin (HSA) nanoparticles, which are subsequently cloaked in red blood cell membranes (RBCMs) for robust immune evasion and prolonged systemic circulation. To achieve active targeting, the nanoparticle surface was functionalized with hyaluronic acid (HA) to selectively bind CD44 receptors, which are heavily overexpressed on RA-driving macrophages and fibroblast-like synoviocytes (FLSs). In vitro evaluations demonstrated significantly enhanced cellular internalization by activating RAW264.7 macrophages and FLS, resulting in the potent suppression of pro-inflammatory cytokines (TNF-α, IL-1β, IL-6) with minimal baseline cytotoxicity. Furthermore, comprehensive in vivo studies using a collagen-induced arthritis (CIA) murine model confirmed that MTX@HSA@M@HA NPs significantly ameliorated joint inflammation, attenuated paw swelling, and rapidly improved functional outcomes compared to free MTX. By synergizing RBCM camouflage with HA-directed active targeting, this nanoplatform maximizes localized therapeutic efficacy while minimizing systemic toxicity, thereby presenting a highly promising and translatable strategy for targeted RA treatment.

## 1. Introduction

Rheumatoid arthritis (RA) is a debilitating chronic autoimmune disease characterized by progressive synovial inflammation, cartilage destruction, and bone erosion, affecting approximately 0.5–1.0% of the global population [1,2]. The complex pathogenesis of RA is fundamentally driven by the extensive infiltration and aberrant activation of inflammatory cells within the synovial joints [3,4,5]. Specifically, macrophages and fibroblast-like synoviocytes (FLSs) proliferate rapidly, leading to pannus formation and the secretion of a complex network of pro-inflammatory cytokines, including tumor necrosis factor-α (TNF-α), interleukin-1 (IL-1), and interleukin-6 (IL-6) [6,7,8]. This localized cellular infiltration and the resulting cytokine storm create a highly aggressive inflammatory microenvironment that dictates the chronic progression of the disease.

Current therapeutic interventions, including nonsteroidal anti-inflammatory drugs (NSAIDs), glucocorticoids (GCs), disease-modifying antirheumatic drugs (DMARDs), and biologics, primarily aim to systemically suppress this inflammatory cascade. However, because these agents lack specificity to the inflamed synovium, they are often hindered by suboptimal localized efficacy, severe systemic toxicity, and off-target adverse effects [9,10,11,12]. To overcome these clinical barriers, nanoparticle-based drug delivery systems have emerged as promising platforms to improve targeted accumulation and pharmacokinetics [13,14,15]. Yet, conventional monovalent targeted nanoparticles are often inadequate for managing the multifactorial heterogeneity of RA; their limited specificity can trigger compensatory immune clearance and drug resistance [16,17]. In contrast, dual-targeted nanoparticles that simultaneously engage multiple pathological pathways offer a superior strategy, synergistically enhancing targeting accuracy, improving therapeutic efficacy, and mitigating systemic side effects [18,19,20,21,22].

**Figure 1:**
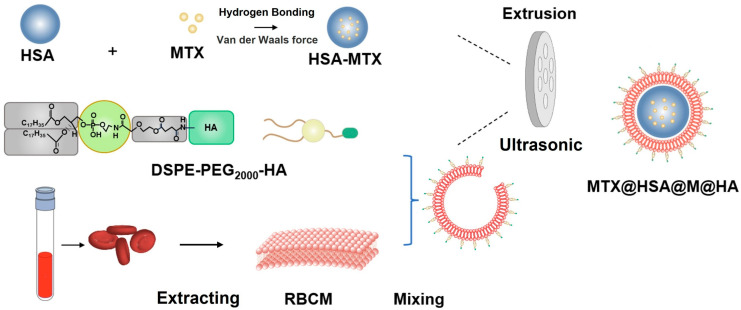
Schematic illustration of the preparation process of dual-targeted, low-immunogenicity nanoparticles.

To construct an ideal dual-targeted biomimetic nanoplatform (MTX@HSA@M@HA NPs), we integrated specific functional materials tailored to the RA microenvironment. Red blood cell membranes (RBCMs) were utilized for their exceptional biocompatibility and low immunogenicity, enabling the nanocarriers to evade immune surveillance and prolong systemic circulation [23,24,25,26,27,28]. For the therapeutic core, human serum albumin (HSA) was selected to encapsulate Methotrexate (MTX), a cornerstone DMARD. HSA efficiently binds MTX via hydrophobic interactions and hydrogen bonding, significantly improving the drug’s solubility and safety profile. To achieve active targeting to the inflamed joints, the RBCM surface was functionalized with 1,2-Distearoyl-sn-glycero-3-phosphoethanolamine-N-[methoxy(polyethylene glycol)-2000]-hyaluronic acid (DSPE-PEG_2000_-HA) (Figure 1). Hyaluronic acid (HA) not only possesses intrinsic anti-inflammatory properties [29,30], but also selectively binds to CD44 receptors, which are highly overexpressed on the critical pathological drivers of RA—macrophages and FLS. RBC membrane coating primarily contributes to immune evasion, whereas HA modification enhances active targeting; their combination is intended to achieve complementary and synergistic effects.

Building upon this rational design, we hypothesize that the MTX@HSA@M@HA NPs will effectively accumulate at the inflamed joints via CD44-mediated active targeting. To verify this, the present study evaluates the cellular uptake and anti-inflammatory efficacy of the nanoplatform in vitro using RAW264.7 macrophages and FLS. Furthermore, we aim to investigate its therapeutic potential in vivo using a collagen-induced arthritis (CIA) rat model, anticipating a significant alleviation of joint swelling, suppression of pro-inflammatory cytokines (TNF-α, IL-1β, IL-6), and restoration of joint function. Therefore, this work focuses on the development and validation of a multifunctional nanoplatform that simultaneously addresses immune evasion, targeting specificity, and therapeutic efficacy, providing a more comprehensive strategy for RA treatment.

## 2. Materials and Methods

### 2.1. Materials

N-hydroxysuccinimide (NHS), dimethyl sulfoxide (DMSO), 1-(3-dimethylaminopropyl)-3-ethylcarbodiimide (EDC), and hyaluronic acid (HA) were purchased from Aladdin (Shanghai, China). DSPE-PEG_2000_-NH_2_ was obtained from Tianjin Puxitang Biomedical Technology Co., Ltd. Red blood cell sedimentation solution, phosphotungstic acid negative staining solution, methotrexate (MTX), and human serum albumin (HSA) were purchased from Solarbio (Beijing, China). DIR dye was purchased from Dibai Biotechnology (Shanghai, China). Lipopolysaccharide (LPS) was obtained from Sigma-Aldrich (St. Louis, MO, USA). Complete and incomplete Freund’s adjuvants (CFA and IFA) were purchased from MedChemexpress (Monmouth Junction, NJ, USA). Bovine type II collagen was obtained from Chondrex (Washington, DC, USA). The CCK-8 assay kit and Calcein-AM/PI Live-Dead Cell Staining Kit were obtained from Beyotime Biotechnology (Shanghai, China).

### 2.2. Experimental Animals

Male Wistar rats (7 weeks old) were obtained from Shengchang Laboratory Technology (Shanghai, China). All animals were maintained under controlled temperature conditions (22–25 °C) with a 12-h light/dark cycle and provided with a standard diet.

### 2.3. Cell Lines and Culture Conditions

Murine macrophage cell line RAW264.7 was purchased from the Cell Bank of the Chinese Academy of Sciences (Shanghai, China) and cultured in DMEM supplemented with 10% fetal bovine serum (FBS). FLS were isolated from the synovial tissues of CIA rats and cultured in DMEM containing 15% FBS and 1% penicillin-streptomycin. All cells were incubated at 37 °C with 5% CO_2_ in a humidified incubator (SERIES 8000W, Thermo Fisher, Waltham, MA, USA).

### 2.4. Extraction and Purification of Red Blood Cell Membranes

Whole blood was collected from the orbital sinus of healthy adult Sprague–Dawley rats using EDTA-containing anticoagulant tubes. The fresh anticoagulated blood was mixed with 1× PBS and rRBC sedimentation solution at a 1:1:1 ratio and allowed to stand at room temperature for 30–40 min. The supernatant was carefully removed, and the precipitated RBCs were washed three times with PBS. The RBCs were subsequently lysed in a hypotonic lysis buffer containing protease inhibitors. The suspension was homogenized three times using an ultrasonic cell disruptor to lyse the RBCs. The lysate was first centrifuged at 2000× *g* for 15 min at 4 °C to remove cell debris, followed by centrifugation of the supernatant at 12,000× *g* for 20 min at 4 °C. The collected RBC membranes were further extruded through polycarbonate membranes and finally resuspended in 1× PBS and stored at −80 °C.

### 2.5. Synthesis of DSPE-PEG2000-HA

DSPE-PEG_2000_-HA was synthesized based on a previously reported protocol [31]. Briefly, 5 mg hyaluronic acid (HA), 25 mg 1-ethyl-3-(3-dimethylaminopropyl) carbodiimide (EDC), and 50 mg N-hydroxysuccinimide (NHS) were dissolved in PBS and stirred for 60 min at room temperature. Subsequently, 25 mg DSPE-PEG_2000_-NH_2_ (Aladdin, Shanghai, China) was added to the reaction mixture and stirred continuously for 24 h. The resulting solution was then dialyzed using a dialysis bag (MWCO 2500, FEIYUBIO, Nantong, China) and lyophilized. The final product was stored at 4 °C for further use.

### 2.6. Preparation of MTX-Loaded Nanoparticles

MTX was first dissolved in DMSO to a concentration of 10 mg/mL, followed by the addition of NHS and EDC. The mixture was stirred for 24 h at room temperature. Meanwhile, a 5 mg/mL solution of HSA was prepared. The MTX solution was slowly added dropwise into the HSA solution under constant stirring, and the reaction was allowed to proceed overnight at room temperature. The resulting mixture was then dialyzed using a dialysis bag (MWCO 3500) against PBS to remove excess MTX, DMSO, and unbound HSA. The dialyzed solution was lyophilized to obtain MTX@HSA nanoparticles, which were stored at 4 °C.

To fabricate the dual-modified nanoparticles, the collected RBCMs were mixed with the synthesized MTX@HSA nanoparticles and DSPE-PEG2000-HA in PBS. The mixture was stirred for 30 min and subsequently extruded through 220 nm polycarbonate membranes using a mini extruder. The dispersion was subjected to probe sonication to achieve homogeneity, resulting in the formation of MTX@HSA@M@HA nanoparticles. Western blotting was employed to evaluate the protein expression profiles of RBCMs.

### 2.7. Preparation and Characterization of Nanoparticles

The particle size and distribution were measured using a Zeta Viewer equipped with a 640 nm laser and a fluorocarbon O-ring sample cell. Samples were injected into the chamber via sterile syringes, and measurements were conducted for 40 s using manual shutter and gain settings. Data analysis was performed using NTA software (version 3.2, Build 127). Dynamic light scattering (DLS) was used to determine the polydispersity index (PDI) and zeta potential. Zeta potential measurements were conducted at 4 mV voltage for 10 cycles using a red laser (675 nm) at a 90° angle. All measurements were performed at 25 °C.

### 2.8. In Vitro Release of MTX from Nanoparticles

A dialysis method was used to evaluate the in vitro release behavior of MTX under simulated physiological conditions. MTX@HSA and MTX@HSA@M@HA nanoparticles were suspended in PBS (pH 7.4, containing 0.5% Tween-80) or in pH 5.0 acetate buffer to mimic the acidic microenvironment of inflamed joints. The suspensions were sealed in dialysis bags (MWCO 3.5 kDa) and placed in 50 mL of the respective release media at 37 °C under gentle shaking. At predetermined time points (0.5, 1, 2, 4, 6, 8, 12, and 24 h), 1 mL of the release medium was withdrawn and replaced with an equal volume of fresh buffer. The absorbance of released MTX was measured using a UV–vis spectrophotometer.

### 2.9. In Vitro Analysis of Nanoparticle Cellular Uptake

RAW264.7 cells were seeded into 24-well plates and incubated overnight. The experimental group was pretreated with LPS (100 ng/mL) for 24 h to induce activation. FLS cells were cultured under standard conditions [32]. Both cell types were incubated with DiR-labeled MTX@HSA@M and MTX@HSA@M@HA nanoparticles for 24 h. After incubation, the cells were washed twice with PBS, fixed with 5% glutaraldehyde, and stained with DAPI and FITC. Fluorescence images were acquired using a confocal laser scanning microscope (A1R, Nikon, Tokyo, Japan), and mean fluorescence intensity (MFI) was quantified for comparative analysis.

### 2.10. Cytotoxicity Assay

RAW264.7 cells were seeded in 96-well plates and incubated for 24 h. Cells were then treated with free MTX, MTX@HSA, or MTX@HSA@M@HA nanoparticles at final concentrations of 50, 100, and 150 μg/mL. After 24 and 72 h of incubation, cell viability was assessed using a CCK-8 assay according to the manufacturer’s protocol [33].

### 2.11. Live/Dead Cell Staining

FLS cells were seeded into 24-well plates at a density of 5 × 10^5^ cells per well. Control wells were treated with DMEM only, while treatment groups received free MTX, MTX@HSA, MTX@HSA@M, or MTX@HSA@M@HA NPs for three days. After treatment, cells were washed with PBS and stained using a Calcein-AM/PI live-dead staining kit (Beyotime, Shanghai, China). Fluorescence microscopy (APX100, OLYMPUS, Tokyo, Japan) was used to visualize live and dead cells.

### 2.12. Induction of CIA Rat Model

To induce collagen-induced arthritis (CIA), male Wistar rats were acclimated for at least 1 week. Rats were then subcutaneously injected at the tail base with 100 µL of type II collagen emulsion in CFA. A booster injection with type II collagen in IFA was administered at day 7 post-initial immunization. The equivalent MTX dose is 20 mg/kg. Clinical signs were monitored from day 0, including body weight, paw thickness, and arthritis scores. The scoring criteria were as follows: 0 = no symptoms; 1 = mild swelling; 2 = moderate swelling confined to joints; 3 = severe swelling extending to digits; 4 = maximal swelling, purplish-red coloration, and joint deformation. The sample size for the animal experiments in this study was 5 rats per group (*n* = 5). To reduce bias, all assessments were performed in a blinded manner. Animal procedures were approved by the Institutional Animal Care and Use Committee of East China University of Science and Technology (ECUST-2025-090).

### 2.13. Micro-CT Analysis

Two days after the final treatment, ankle joints were collected and fixed in 4% paraformaldehyde. Micro-CT scanning (NMC-200, PINGSENG Healthcare, Jiangsu, China) was performed with the following parameters: 80 kV tube voltage, 0.06 mA current, 35 μm resolution, and 20 frames/s scan speed. Reconstruction was performed using Recon (v1.7.4.2) software, and quantitative analysis of bone density and trabecular architecture within the region of interest (ROI) were conducted using Avatar (v1.0) software.

### 2.14. Statistical Analysis

All data were analyzed using GraphPad Prism 10.1.2. Results are presented as mean ± standard error of the mean (SEM). One- or two-way analysis of variance (ANOVA) was used for statistical comparison among multiple groups, followed by Tukey’s post hoc test for multiple comparison correction. The *p*-value of <0.05 was considered as a significant difference between groups (* *p* < 0.05, ** *p* < 0.01, *** *p* < 0.005, **** *p* < 0.001).

## 3. Results

### 3.1. Characterization of Nanoparticles

**Figure 2:**
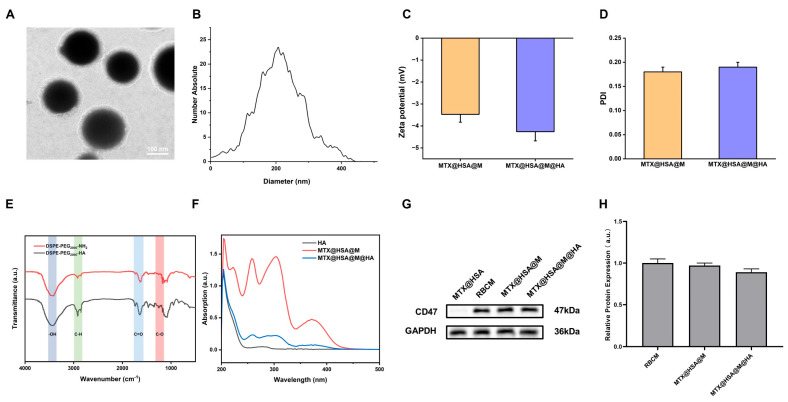
Structural and compositional characterization of MTX@HSA@M@HA NPs. (**A**) TEM images showing the typical core–−shell structure. (**B**) Particle size distribution measured by NTA. (**C**) Zeta potential of MTX@HSA@M and MTX@HSA@M@HA NPs. (**D**) PDI values of the nanoparticles. (**E**) FT-IR spectra confirming the synthesis of DSPE-PEG_2000_-HA via characteristic amide bond peaks. (**F**) UV-Vis spectra showing the characteristic absorption peaks of both HA and MTX@HSA@M. (**G**) Western blot analysis of CD47 protein expression on MTX@HSA NPs, RBCM, MTX@HSA@M, and MTX@HSA@M@HA NPs. (**H**) Quantitative analysis of relative expression levels.

TEM images revealed that MTX@HSA@M@HA nanoparticles exhibited a typical core–shell structure, indicating successful and complete coating with RBCM and HA (Figure 2A). Macroscopic photographs (Supplementary Materials Figure S1) and TEM images (Supplementary Materials Figure S2) show that HSA NPs are transparent (34.3 ± 6.4 nm), while MTX@HSA NPs are pale yellow with an increased size (94.4 ± 10.4 nm). Nanoparticle tracking analysis (NTA) showed that the average particle size of MTX@HSA@M@HA NPs was 214.5 ± 2.3 nm, with a near-normal distribution profile (Figure 2B). Dynamic light scattering (DLS) analysis demonstrated that the zeta potential and polydispersity index (PDI) of MTX@HSA@M NPs were –3.47 ± 0.36 mV and 0.18 ± 0.01, respectively, while those of MTX@HSA@M@HA NPs were –4.46 ± 0.42 mV and 0.19 ± 0.01, respectively. These results indicate that surface modification with HA did not significantly affect the overall surface charge or colloidal stability of the nanoparticles, which maintained good dispersion and stability (Figure 2C,D). DSPE-PEG_2000_-NH2 was conjugated with HA via amide bond formation between the amino group of DSPE-PEG_2000_-NH2 and the NHS ester-activated carboxyl group of HA. The presence of a C=O stretching vibration peak at 1630–1680 cm^−1^ in the FT-IR spectrum confirmed the successful synthesis of DSPE-PEG_2000_-HA (Figure 2E). In the UV–vis absorption spectra, MTX@HSA@M@HA NPs exhibited characteristic absorption peaks corresponding to both HA and MTX@HSA@M, indicating that the optical properties of both components were retained at the molecular level (Figure 2F). The encapsulation efficiency (EE) reached a maximum of 92.9 ± 3.5% with a drug loading (DL) of 4.8 ± 0.4% at an HSA:MTX ratio of 10:1. Similarly, at an HSA:M ratio of 1:1, EE peaked at 93.2 ± 3.7%, with an increased DL of 5.5 ± 0.4% (Supplementary Materials Figure S3). Western blot analysis further demonstrated high expression of CD47 on the surfaces of RBCM vesicles, MTX@HSA@M, and MTX@HSA@M@HA NPs, confirming the successful RBC membrane coating. This feature is beneficial for reducing immunogenicity and prolonging systemic circulation time (Figure 2G,H).

### 3.2. In Vitro Characterization of Nanoparticles

**Figure 3:**
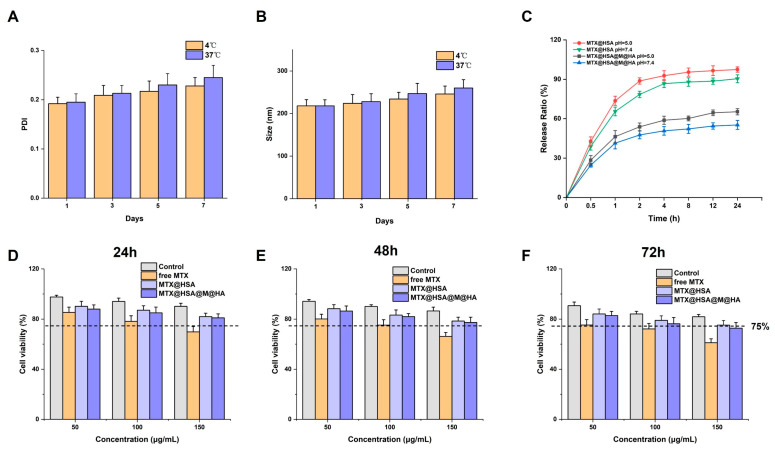
In vitro characterization of MTX@HSA@M@HA NPs. Stability of (**A**) PDI and (**B**) particle size at 4 °C and 37 °C over 7 days. (**C**) In vitro drug release profiles of MTX@HSA and MTX@HSA@M@HA NPs under pH 5.0 and 7.4 conditions. (**D**–**F**) Cell viability of Control, free MTX, MTX@HSA, and MTX@HSA@M@HA groups measured by CCK-8 assay from 24 to 72 h.

The particle size stability of nanoparticles is a critical parameter for evaluating their overall stability. At both 4 °C and 37 °C, a gradual increase in particle size was observed over a 1–7 day period, with slightly faster growth at 37 °C compared to 4 °C. However, the difference was not statistically significant, suggesting that temperature had a limited impact on particle size variation. Combined with the PDI values, no substantial changes in particle size or size distribution were detected under either storage condition during the observation period, indicating good short-term stability of the nanoparticle system (Figure 3A,B). Under simulated RA inflammatory conditions (pH = 5.0), MTX@HSA NPs exhibited a rapid initial release, reaching 73.6% within the first hour, followed by a slower sustained release phase that approached complete drug release. Compared to the release at pH = 7.4, the cumulative release at pH = 5.0 was higher. MTX@HSA@M@HA NPs also demonstrated accelerated early release under acidic conditions, with 46.3% of MTX released within the first hour, eventually reaching 65.3%. Although both systems showed increased release under acidic pH, the multilayered structure of RBC membrane and HA imparted sustained release characteristics. At both pH 5.0 and 7.4, MTX@HSA@M@HA NPs exhibited a slower and lower cumulative release compared to MTX@HSA NPs, indicating that the multilayer modifications effectively regulated MTX release and prolonged its therapeutic window (Figure 3C).

Over an extended exposure period from 24 to 72 h, free MTX exhibited a time-dependent increase in cytotoxicity, with progressively decreasing cell viability, consistent with a typical time–toxicity accumulation effect. In contrast, cells treated with MTX@HSA NPs and MTX@HSA@M@HA NPs showed significantly higher viability at all time points and drug concentrations compared to the free MTX group. These findings confirm the protective role of the nanoparticle carrier system in mitigating MTX-induced cytotoxicity, thereby enhancing cellular tolerance (Figure 3D–F).

### 3.3. In Vivo Cellular Uptake of Nanoparticles

**Figure 4:**
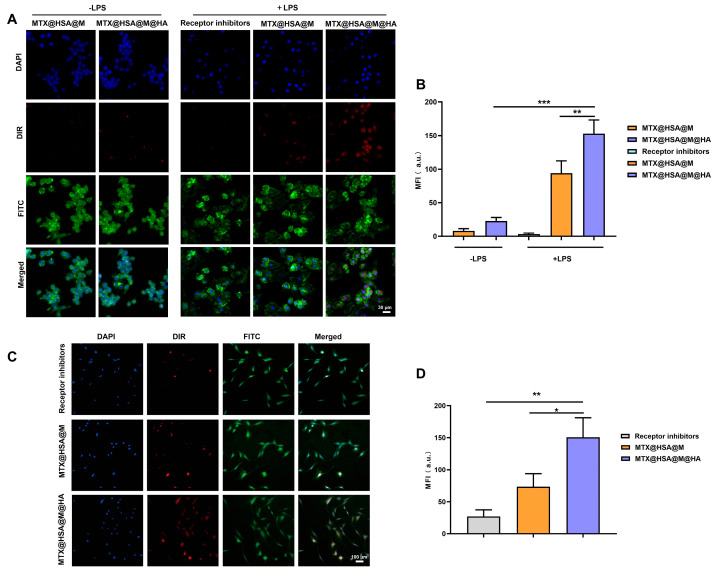
Cellular uptake of MTX@HSA@M and MTX@HSA@M@HA NPs in vitro. (**A**) Fluorescence images and (**B**) MFI of RAW264.7 cells with or without LPS stimulation. (**C**) Fluorescence images and (**D**) MFI of FLS cells. Enhanced uptake of MTX@HSA@M@HA NPs is observed, especially after receptor activation or in presence of HA targeting (* *p* < 0.05, ** *p* < 0.01, *** *p* < 0.005).

To investigate the cellular targeting and uptake efficiency of nanoparticles by RAW264.7 and FLS cells, fluorescence imaging was employed to track the intracellular distribution of MTX@HSA@M and MTX@HSA@M@HA nanoparticles. Upon LPS stimulation, RAW264.7 cells exhibited upregulated expression of the CD44 receptor. In the absence of LPS stimulation, both nanoparticle formulations showed weak DiR-associated red fluorescence signals within cells, indicating minimal uptake under basal conditions. However, following LPS stimulation, MTX@HSA@M@HA nanoparticles exhibited significantly enhanced intracellular red fluorescence, suggesting increased uptake (Figure 4A). Moreover, fluorescence intensity in the receptor inhibitor-treated group was markedly lower than that of the MTX@HSA@M@HA group, further supporting CD44-mediated endocytosis. Quantitative analysis of MFI revealed that the MFI value of the MTX@HSA@M@HA group was substantially higher than that of the MTX@HSA@M group under LPS stimulation (Figure 4B).

Similarly, in FLS cells (Figure 4C), the MTX@HSA@M@HA group exhibited significantly stronger red fluorescence compared to the MTX@HSA@M group, indicating greater cellular uptake. Quantification of MFI confirmed that the MTX@HSA@M@HA group had a significantly higher MFI than both the MTX@HSA@M group and the receptor inhibitor group (Figure 4D), demonstrating enhanced uptake efficiency. FLS cells are also known to express high levels of CD44 receptors, which facilitate targeted nanoparticle internalization via CD44-mediated endocytosis. Collectively, these results indicate that MTX@HSA@M@HA nanoparticles possess robust cellular targeting capabilities and are capable of dual targeting of both LPS-activated RAW264.7 macrophages and FLS cells.

### 3.4. In Vitro Anti-Inflammatory Effects of Nanoparticles

**Figure 5:**
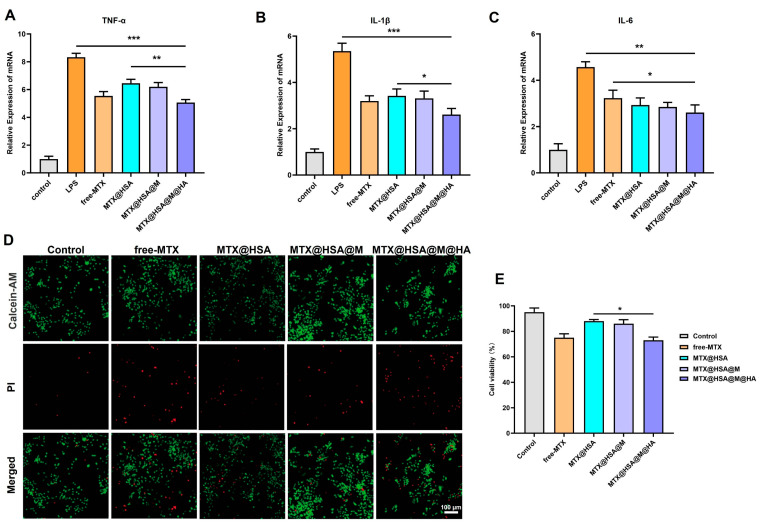
In vitro anti-inflammatory effects of MTX@HSA@M@HA NPs on RAW264.7 macrophages and FLS cells. Quantitative PCR analysis of pro-inflammatory cytokines: (**A**) TNF-α, (**B**) IL-1β, and (**C**) IL-6 mRNA expression levels. (**D**) Live/dead staining images and (**E**) cell viability assays of FLS cells after treatment, showing inhibition of abnormal proliferation by MTX@HSA@M@HA NPs (* *p* < 0.05, ** *p* < 0.01, *** *p* < 0.005).

qPCR analysis demonstrated that MTX@HSA@M@HA nanoparticles exhibited potent anti-inflammatory activity in RAW264.7 cells in vitro (Figure 5A–C). Compared to the LPS-stimulated group, all treatment groups showed varying degrees of inhibition on the mRNA expression levels of pro-inflammatory cytokines TNF-α, IL-1β, and IL-6. Among them, the MTX@HSA@M@HA group showed the most significant suppression, outperforming the free MTX, MTX@HSA, and MTX@HSA@M groups. These findings suggest that HA modification not only enhances the nanoparticles’ targeting capability toward inflammatory macrophages, but also likely facilitates more efficient intracellular delivery of the drug, thereby synergistically improving anti-inflammatory efficacy.

In RA, abnormal proliferation of FLSs contributes to synovial hyperplasia and thickening. These activated FLSs secrete cytokines and inflammatory mediators that exacerbate immune cell infiltration and joint destruction. Therefore, inhibiting FLS proliferation is essential for mitigating synovial inflammation and preserving joint structure and function. Live/dead staining results (Figure 5D,E) showed the weakest fluorescence signal in the control group and the strongest in the MTX@HSA@M@HA NPs group. Notably, MTX@HSA@M@HA NPs exerted a significant inhibitory effect on FLS proliferation, whereas MTX@HSA NPs showed minimal suppression. These results indicate that MTX@HSA@M@HA nanoparticles can effectively attenuate RA progression by inhibiting FLS proliferation.

### 3.5. In Vivo Therapeutic Efficacy of Nanoparticles

**Figure 6:**
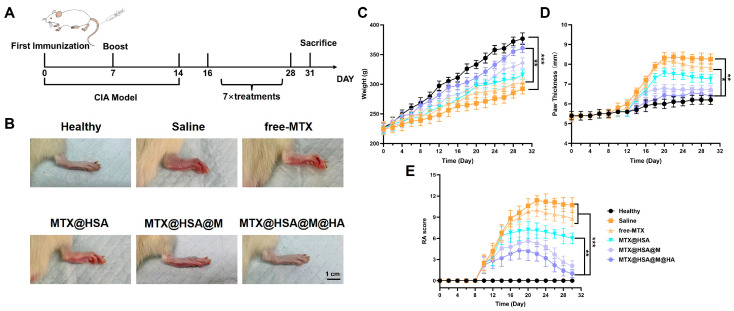
In vivo therapeutic efficacy of MTX@HSA@M@HA NPs in RA rat model. (**A**) Treatment schedule illustrating intravenous injections every other day starting on day 16 for a total of 7 doses. (**B**) Representative images of rat paws on day 30 across different groups. (**C**) Body weight changes; (**D**) paw thickness measurements; (**E**) clinical RA scores. MTX@HSA@M@HA group showed superior therapeutic outcomes compared to other treatment groups (* *p* < 0.05, ** *p* < 0.01, *** *p* < 0.005).

On day 16, rats in the treatment groups received tail vein injections every other day for a total of seven administrations (Figure 6A). Representative images of the rats’ hind paws on day 30 revealed distinct therapeutic outcomes across groups. The Healthy group exhibited normal paw appearance with no visible swelling or erythema. In contrast, the Saline group showed pronounced redness and swelling, characteristic of RA pathology. The free MTX group demonstrated modest alleviation of inflammation. The MTX@HSA group exhibited a certain degree of anti-inflammatory effect, which may be attributed to improved drug efficacy via the nanocarrier. The MTX@HSA@M group, benefitting from RBCM-assisted delivery, showed more pronounced inflammation relief. Notably, the MTX@HSA@M@HA group exhibited nearly normal paw morphology with minimal swelling or discoloration, indicating superior targeted anti-inflammatory efficacy (Figure 6B).

Therapeutic efficacy was further evaluated by monitoring body weight, paw thickness, and RA clinical scores over time (Figure 6C–E). Rats in the Healthy group showed steady weight gain and maintained normal paw thickness and RA scores. In contrast, the Saline group displayed classic RA manifestations, including slow weight gain, marked paw swelling, and elevated RA scores. Among the treatment groups, free MTX demonstrated limited anti-inflammatory effects, likely due to its lack of targeted delivery. MTX@HSA nanoparticles, leveraging the HSA nanocarrier, partially alleviated inflammation by improving drug distribution. MTX@HSA@M nanoparticles further enhanced therapeutic efficacy through RBCM-mediated delivery. Most notably, MTX@HSA@M@HA nanoparticles, incorporating HA for active targeting in addition to RBCM coating, achieved substantial improvements in treatment outcomes—evident in body weight recovery, reduced paw swelling, and decreased RA scores. Collectively, these results underscore that the synergistic design of RBCM and HA-modified nanoparticles markedly improves MTX therapeutic performance and offers a promising strategy for precise RA treatment and the development of intelligent drug delivery systems.

### 3.6. Micro-CT Imaging and Bone Parameter Analysis of Rat Ankle Joints

**Figure 7:**
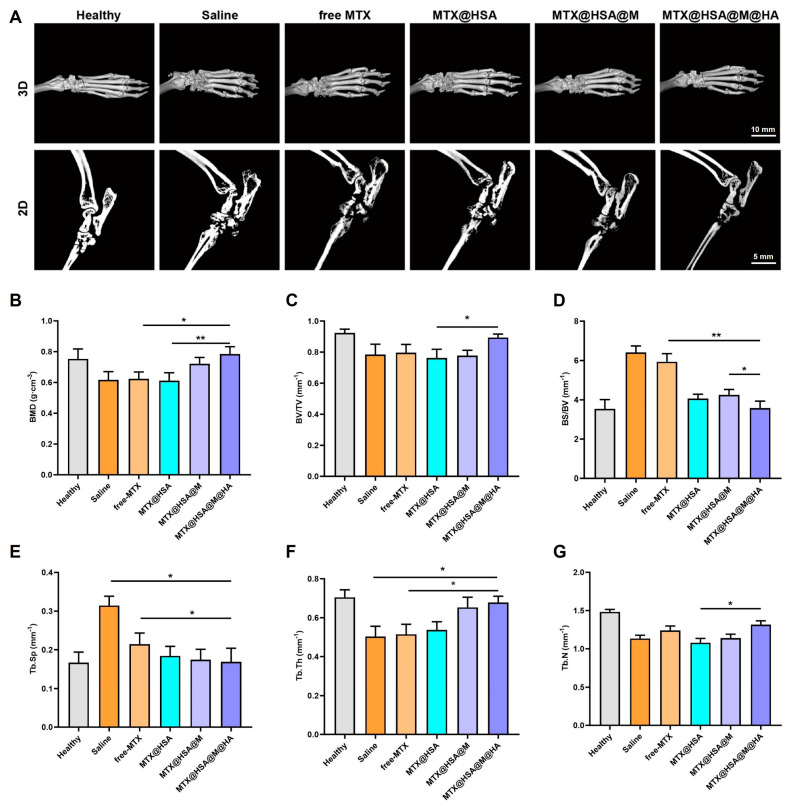
Micro-CT analysis and bone parameter quantification of ankle joints from treated rats. (**A**) Representative 3D Micro-CT images on day 30 showing bone structure restoration. Quantitative evaluation of bone parameters including (**B**) BMD, (**C**) BV/TV, (**D**) BS/BV, (**E**) Tb.Sp, (**F**) Tb.Th, and (**G**) Tb.N. MTX@HSA@M@HA NPs treatment significantly ameliorated bone damage induced by RA. Statistical significance was compared with the Saline group (* *p* < 0.05, ** *p* < 0.01).

Micro-CT was employed to visualize the ankle joints of rats in each group. In the Healthy group, the bone architecture was intact with normal joint morphology, indicating a well-maintained physiological state. In contrast, rats in the Saline group exhibited severe joint deformities, including bone erosion, narrowed joint space, and partial joint fusion, resulting from RA-induced inflammation. The free-MTX group showed slight improvement, but its therapeutic efficacy was limited due to the lack of targeted delivery. The MTX@HSA group demonstrated enhanced inhibition of bone damage, attributed to improved drug distribution via the HSA-based nanocarrier. The MTX@HSA@M group, with prolonged circulation conferred by RBCM coating, further enhanced drug delivery efficiency and mitigated skeletal abnormalities. Remarkably, the MTX@HSA@M@HA group, utilizing HA for active targeting of CD44 receptors, achieved substantial drug accumulation at inflamed joints. This resulted in nearly complete restoration of bone architecture, highlighting the superior therapeutic potential of this system in ameliorating RA-associated bone destruction (Figure 7A).

Quantitative analysis of bone mineral density and trabecular bone parameters further confirmed the therapeutic differences among groups (Figure 7B–G). From the Saline group to the free-MTX, MTX@HSA, MTX@HSA@M, and finally the MTX@HSA@M@HA NP group, there was a progressive increase in BMD, trabecular thickness, and trabecular number, alongside a reduction in trabecular separation. In addition, the bone surface area-to-volume ratio gradually returned to normal levels, indicating suppression of arthritic bone destruction and promotion of bone regeneration across treatment groups. These outcomes may be attributed to the synergistic modifications with HA and RBCM, which confer enhanced biocompatibility, immune evasion, and inflammation-targeting capacity to the nanoparticles, thereby maximizing their therapeutic efficacy. In contrast, free MTX and unmodified nanoparticles exhibited limited effectiveness in restoring bone structure, underscoring the need for targeted delivery strategies in the treatment of inflammation-induced bone lesions. The results demonstrated a reduction in joint inflammation and improvements in clinical and histological indicators.

## 4. Discussion

We developed a nanoparticle system, MTX@HSA@M@HA NPs, comprising MTX loaded onto HSA as a carrier, encapsulated with RBCM, and surface-modified with DSPE-PEG_2000_-HA. The resulting nanoparticles exhibited uniform size and demonstrated satisfactory stability in vitro. They showed targeted uptake by LPS-activated RAW264.7 macrophages and FLS, effectively reducing the expression of pro-inflammatory cytokines TNF-α, IL-1β, and IL-6 in activated RAW264.7 cells. In a CIA rat model, MTX@HSA@M@HA NPs significantly alleviated joint inflammation, reduced swelling, and improved joint function, demonstrating pronounced therapeutic efficacy. These findings indicate that this nano-delivery system offers substantial advantages in enhancing drug targeting and therapeutic efficiency, providing a promising new strategy for the treatment of rheumatoid arthritis.

In recent years, with the rapid advancement of nanomedicine and biomimetic materials science, targeted nanocarrier systems designed based on disease-specific microenvironmental characteristics have emerged as a promising strategy for the treatment of RA. Inflamed joints in RA are typically characterized by enhanced vascular permeability, extensive infiltration of inflammatory cells, and a pathological microenvironment featuring acidic pH, elevated levels of ROS, and increased enzymatic activity. These pathological features provide favorable conditions for the passive accumulation of nanomedicines as well as stimulus-responsive drug release [34,35]. On this basis, the incorporation of biomimetic strategies and active targeting mechanisms has the potential to further improve drug delivery efficiency and therapeutic specificity at inflamed sites.

Biomimetic targeted nanocarrier systems have attracted increasing attention in RA therapy. Among these approaches, cell membrane-based biomimetic strategies have demonstrated distinct advantages owing to their excellent biocompatibility and immune evasion properties. Membranes derived from red blood cells, macrophages, neutrophils, and platelets have been extensively employed to construct biomimetic nanocarriers that recapitulate key biological functions of native cells [36]. Previous studies have shown that red blood cell membrane coated nanoparticles can effectively prolong systemic circulation and reduce clearance by the mononuclear phagocyte system, thereby enhancing drug accumulation at inflamed tissues [37]. In parallel, nanocarriers derived from immune cell membranes such as macrophages or neutrophils retain multiple inflammation-associated chemotactic and adhesion molecules, which enable active homing to inflamed sites and participation in inflammatory regulation, highlighting their therapeutic potential in RA [38,39].

Building upon these advances, the integration of cell membrane biomimicry with ligand-mediated active targeting is regarded as a more precise drug delivery strategy. Within the inflammatory microenvironment of RA, multiple receptors are aberrantly overexpressed, among which CD44, a principal receptor for hyaluronic acid, is highly expressed on activated macrophages and fibroblast like synoviocytes [40]. Accordingly, hyaluronic acid has been widely employed as a targeting ligand in RA-oriented nanocarrier systems. Accumulating evidence indicates that hyaluronic acid-modified nanoparticles can significantly enhance retention at inflamed joints and promote cellular uptake, thereby improving anti-inflammatory efficacy while reducing systemic adverse effects. Therefore, the combination of biomimetic cell membrane coating and hyaluronic acid-mediated targeting provides a solid theoretical basis for precision therapy in RA.

In addition, multiresponsive nanocarrier systems represent an important developmental direction in RA nanotherapy [41]. The inflammatory joint microenvironment is commonly associated with decreased pH, elevated ROS levels, and abnormal activation of-specific enzymes such as matrix metalloproteinases. Nanocarriers designed to respond to these pathological cues, including pH-responsive, ROS-responsive, or enzyme-responsive systems, enable site-specific and on-demand drug release [42]. This design strategy further enhances therapeutic efficacy while minimizing off-target toxicity. Several studies have demonstrated that multistimuli-responsive designs markedly improve spatiotemporal control over drug release and show considerable potential for the regulation of chronic inflammation in RA.

Despite the promising therapeutic outcomes, several limitations should be acknowledged. First, the current study focuses on short-term efficacy in an acute RA model, and long-term safety, repeated dosing effects, and potential organ accumulation remain to be investigated. Second, comprehensive immunological evaluation is required to fully assess the immune response upon prolonged administration. Furthermore, large-scale production, batch-to-batch consistency, and regulatory considerations represent additional challenges for clinical translation. In addition, the interaction between RBC membrane-coated nanoparticles and the immune system is complex and may involve multiple pathways, including macrophage recognition, complement activation, and protein corona formation. A deeper mechanistic understanding of these processes will be essential for optimizing biomimetic nanocarrier design in future studies.

It should be noted that only male rats were used in this study, which is a common practice in preclinical RA models to minimize variability arising from sex hormone fluctuations in females. However, sex differences have been reported in RA pathogenesis and immune responses. Therefore, future studies including both male and female animals are warranted to further validate the therapeutic efficacy and broaden the translational relevance of the proposed nanoplatform.

Collectively, nanotherapeutic strategies for RA are progressively evolving from conventional single function drug delivery systems toward multifunctional, biomimetic, and precision-regulated platforms [43]. Biomimetic targeting systems improve the in vivo fate of nanomedicines by mimicking native cellular behaviors. Cell membrane coating strategies confer prolonged circulation, reduced immune clearance, and enhanced accumulation at inflamed sites. Compared with single-targeted delivery systems, the dual-targeted strategy can effectively break through the key bottlenecks prevailing in current nanotherapies for rheumatoid arthritis, including insufficient targeting specificity and limited therapeutic efficacy. Multiresponsive modifications enable spatiotemporally controlled drug release within the inflammatory microenvironment. Within this context, the MTX@HSA@M@HA nanoparticle system developed in the present study integrates red blood cell membrane-mediated immune evasion with hyaluronic acid-based active targeting of inflammatory cells. This system achieves coordinated responsiveness and precise intervention within the RA inflammatory milieu. Furthermore, it demonstrates pronounced anti-inflammatory efficacy in both in vitro and in vivo models and provides valuable insights into the rational design and clinical translation of biomimetic multifunctional nanoplatforms for precision therapy in RA.

## 5. Conclusions

We successfully engineered a biomimetic targeted nano-delivery system, MTX@HSA@M@HA NPs. The fabricated nanoparticles exhibited uniform size distribution, favorable in vitro stability, and specific targeting capabilities toward LPS-activated RAW264.7 macrophages and FLS cells. In vitro experiments demonstrated potent suppression of pro-inflammatory cytokines (TNF-α, IL-1β, IL-6). In a CIA murine model of RA, the nano-formulation significantly ameliorated joint inflammation, attenuated paw swelling, and improved functional outcomes, demonstrating superior therapeutic efficacy compared to conventional methotrexate (MTX) administration.

The exceptional therapeutic performance of MTX@HSA@M@HA NPs can be attributed to the synergistic integration of functional design elements. The RBCM coating prolongs systemic circulation time and mitigates immune clearance through biomimetic camouflage, whereas HA surface modification enables active targeting of CD44-overexpressing inflammatory cells within arthritic joints. HA-mediated CD44 targeting is a well-established mechanism, and the enhanced uptake observed here is consistent with previously reported HA-functionalized nanocarriers under inflammatory conditions. This dual-targeting strategy enhances localized drug accumulation while minimizing systemic off-target toxicity. These findings confirm that the combination of biomimetic cell membrane coating with ligand-mediated active targeting represents an optimized approach for RA nano-therapy.

## Data Availability

The original contributions presented in this study are included in the article/supplementary material. Further inquiries can be directed to the corresponding author.

## Supplementary Materials

The following supporting information can be downloaded at: https://www.mdpi.com/article/10.3390/jfb17050228/s1, Figure S1: Compared photos of different samples: (A) HSA NPs; (B) MTX@HSA NPs; (C) MTX@HSA@M@HA NPs; Figure S2: Compared TEM images: (A) HSA NPs and (B) MTX@HSA NPs. The particle size of HSA NPs is 34.3 ± 6.4 nm. The particle size of MTX@HSA NPs is 94.4 ± 10.4 nm; Figure S3: Effects of HSA:MTX (A) and HSA:M (B) on EE and DL values when the mass ratio of HSA to MTX reached 10:1, the encapsulation efficiency (EE) achieved its maximum value of 92.9 ± 3.5%, with a corresponding drug loading (DL) of 4.8 ± 0.4%. Adjusting the ratio of HSA to membrane material (M), the encapsulation efficiency (EE) reached a peak value of 93.2 ± 3.7% at an HSA:M ratio of 1:1, accompanied by an increase in drug loading (DL) to 5.5 ± 0.4%.

## Abbreviations

The following abbreviations are used in this manuscript:

RA	Rheumatoid Arthritis
MTX	Methotrexate
HSA	Human Serum Albumin
RBCM	Red Blood Cell Membrane
CIA	Collagen-Induced Arthritis
FLS	Fibroblast-Like Synoviocytes
LPS	Lipopolysaccharide
TNF-α	Tumor Necrosis Factor-alpha
IL-1β	Interleukin-1beta
IL-6	Interleukin-6
CCK-8	Cell Counting Kit-8
DLS	Dynamic Light Scattering
NTA	Nanoparticle Tracking Analysis
TEM	Transmission Electron Microscopy
FT-IR	Fourier Transform Infrared Spectroscopy
PDI	Polydispersity Index
EDC	1-Ethyl-3-(3-dimethylaminopropyl)carbodiimide
NHS	N-Hydroxysuccinimide
DMSO	Dimethyl Sulfoxide
PBS	Phosphate-Buffered Saline
FBS	Fetal Bovine Serum
DMEM	Dulbecco’s Modified Eagle Medium
FITC	Fluorescein Isothiocyanate
DAPI	4′,6-Diamidino-2-Phenylindole
MFI	Mean Fluorescence Intensity
Micro-CT	Micro-Computed Tomography
BMD	Bone Mineral Density
BV/TV	Bone Volume/Total Volume
BS/BV	Bone Surface/Bone Volume
Tb.Sp	Trabecular Separation
Tb.Th	Trabecular Thickness
Tb.N	Trabecular Number
NSAIDs	Non-Steroidal Anti-Inflammatory Drugs
GCs	Glucocorticoids
DMARDs	Disease-Modifying Antirheumatic Drugs
