# Supplementary material for: Therapeutic Efficacy of Dual-Targeting Nanoparticles with Low Immunogenicity in the Treatment of Rheumatoid Arthritis
Source: J Funct Biomater. 2026 May 6;17(5):228. doi: 10.3390/jfb17050228 (PMC13208255; doi:10.3390/jfb17050228)
Supplement: Supplementary file 1 [file jfb-17-00228-s001.zip › jfb-4270545-supplementary.pdf]

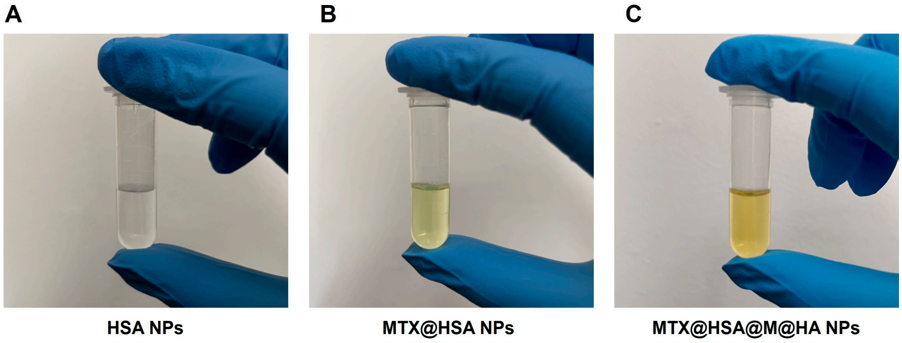

Figure S1. Compared photos of different samples: (A) HSA NPs; (B) MTX@HSA NPs; (C) MTX@HSA@M@HA NPs.

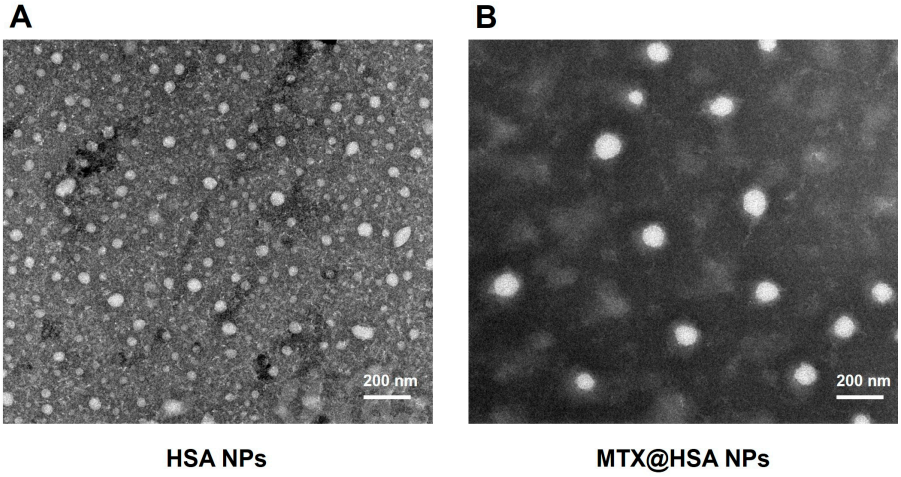

Figure S2. Compared TEM images: (A) HSA NPs and (B) MTX@HSA NPs. The particle size of HSA NPs is 34.3±6.4 nm. The particle size of MTX@HSA NPs is 94.4±10.4 nm.

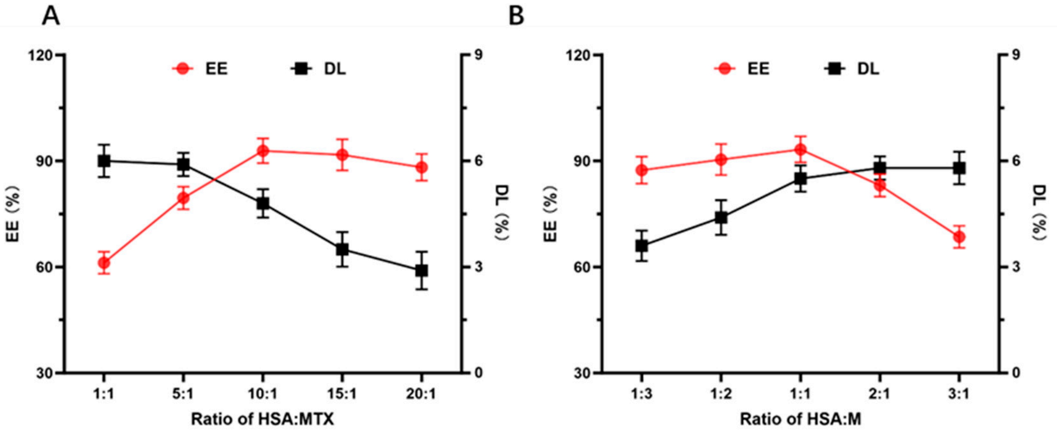

Figure S3. Effects of HSA:MTX (A) and HSA:M (B) on EE and DL values when the mass ratio of HSA to MTX reached 10:1, the encapsulation efficiency (EE) achieved its maximum value of 92.9 ± 3.5%, with a corresponding drug loading (DL) of 4.8 ± 0.4%. Adjusting the ratio of HSA to membrane material (M), the encapsulation efficiency (EE) reached a peak value of 93.2 ± 3.7% at an HSA:M ratio of 1:1, accompanied by an increase in drug loading (DL) to 5.5 ± 0.4%.
